# CSL-Tox: an open-source analytical framework for the comparison of short-term and long-term toxicity end points and assessing the need of chronic studies in drug development

**DOI:** 10.1038/s41598-023-41899-4

**Published:** 2023-09-08

**Authors:** Doha Naga, Smaragda Dimitrakopoulou, Sonia Roberts, Elisabeth Husar, Susanne Mohr, Helen Booler, Eunice Musvasva

**Affiliations:** 1 Roche Pharma Research & Early Development, Roche Innovation Center Basel, F. Hoffmann-La Roche Ltd., Basel, Switzerland; 2 Department of Pharmaceutical Sciences, University of Vienna, Vienna, Austria; 3 Department of Biosystems Science and Engineering, ETH Zurich, Basel, Switzerland

**Keywords:** Toxicology, Preclinical research

## Abstract

In-vivo toxicity assessment is an important step prior to clinical development and is still the main source of data for overall risk assessment of a new molecular entity (NCE). All in-vivo studies are performed according to regulatory requirements and many efforts have been exerted to minimize these studies in accordance with the (Replacement, Reduction and Refinement) 3Rs principle. Many aspects of in-vivo toxicology packages can be optimized to reduce animal use, including the number of studies performed as well as study durations, which is the main focus of this analysis. We performed a statistical comparison of adverse findings observed in 116 short-term versus 78 long-term in-house or in-house sponsored Contract Research Organizations (CRO) studies, in order to explore the possibility of using only short-term studies as a prediction tool for the longer-term effects. All the data analyzed in this study was manually extracted from the toxicology reports (in PDF formats) to construct the dataset. Annotation of treatment related findings was one of the challenges faced during this work. A specific focus was therefore put on the summary and conclusion sections of the reports since they contain expert assessments on whether the findings were considered adverse or were attributed to other reasons. Our analysis showed a general good concordance between short-term and long-term toxicity findings for large molecules and the majority of small molecules. Less concordance was seen for certain body organs, which can be named as “target organ systems’ findings”. While this work supports the minimization of long-term studies, a larger-scale effort would be needed to provide more evidence. We therefore present the steps performed in this study as an open-source R workflow for the Comparison of Short-term and Long-term Toxicity studies (CSL-Tox). The dataset used in the work is provided to allow researchers to reproduce such analysis, re-evaluate the statistical tools used and promote large-scale application of this study. Important aspects of animal research reproducibility are highlighted in this work, specifically, the necessity of a reproducible adverse effects reporting system and utilization of the controlled terminologies in-vivo toxicology reports and finally the importance of open-source analytical workflows that can be assessed by other scientists in the field of preclinical toxicology.

## Introduction

Adverse drug reactions (ADRs) represent one of the major causes of drug attrition^1–4^ and have previously caused several market withdrawals^5–8^. With the purpose of identification and elimination of such hazardous adverse events in the development of new medicines, testing starts prior to studies in animals; safety pharmacology (SP) and in vitro studies are followed by in vivo toxicity studies prior to entry in humans^9–12^. In vivo toxicity studies are pivotal to support clinical studies at all stages up to registration. The required non-clinical safety package varies among molecule classes, but the principles are similar^13–15^. One of the main purposes of in vivo toxicity studies is identification of the target organ specific toxicities, providing a safety margin and exploring the reversibility of the adverse events observed. This information is used to estimate an initial safe starting dose and dose range for human trials and to identify parameters for clinical monitoring of potential adverse effects. Investigation of several parameters that cannot be obtained in clinical trials, such as macroscopic and microscopic findings, help in the identification of toxicity. They also help in the determination of the highest dose that does not produce a significant increase in adverse events and does not raise any safety concerns, defined as the No Observed Adverse Effect Level (NOAEL)^16^.

In-vivo toxicity experiments are performed in accordance with the replace, refine and reduce (3Rs) principles^17,18^. Sparrow et al. reviewed the study designs of cross-company toxicity studies and highlighted the factors affecting the number of animals used, such as the general design of the toxicology program and the use of control groups^19^. They proposed case-specific adjustments in study designs that allow for minimization of animal use. The use of virtual control groups has also been proposed, using historical data to reduce the number of animals in contemporaneous control groups^20^. Other efforts proposed the use of only one species in long-term toxicity studies^21^.^.^All these efforts contribute to the minimization of the number of animals used in toxicity studies.

**Figure 1:**
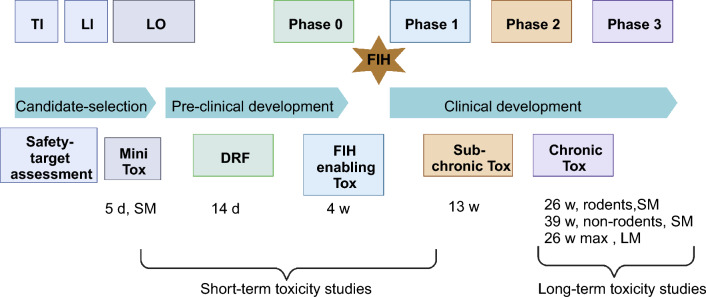
Toxicological testing of new molecular entities in the different stages of drug discovery and development. *TI* Target identification, *LI* Lead identification, *LO* Lead optimization, *Phase 0* represents the start of the preclinical development phase where animal studies are initiated, *d* days, *w* week, First in-human enabling studies (*FIH*) is initiated at *Phase 1* and terminates at *Phase 3* (phase 1 to phase 3 represents the clinical development stage), *DRF* represents the Dose range finding studies, *SM* Small molecules, *LM* Large Molecules and *max* maximum.

Reduction of the number of studies performed is another aspect serving the 3Rs principle and is the focus of this work. Toxicity can become manifest either after a short time and/or only after repeated exposure to the drug throughout longer durations. The recommended duration of repeated-dose toxicity studies is usually related to the duration, therapeutic indication and scope of the proposed clinical trial. Figure 1 represents a general overview of the different types of toxicity studies performed during the drug development process and the corresponding durations. These durations are dependent on the clinical program and the type of molecule and might vary between pharmaceutical companies. Generally, repeated-dose toxicity studies for a minimum duration of 2 weeks would generally support any clinical development trial up to 2 weeks in duration. Clinical trials of longer duration should be supported by repeated-dose toxicity studies of at least equivalent duration, for example, 6-month rodent and 9-month non-rodent studies generally support dosing for longer than 6 months in clinical trials (see ICH M3 guideline)^13^. Toxicity studies can be classified into short-term studies e.g. acute toxicity, sub-chronic studies that generally last for 1 to 3 months and long-term studies e.g. chronic studies that may last up to 12 months. Generally, long-term studies can indicate the progression of findings that were either not observed in shorter studies or were of low incidence, or less severe and therefore not considered adverse. However, this might not always be the case and sometimes no new adverse events are observed upon increasing the study duration. This might provide an opportunity to remove some of the long-term studies, therefore reducing the number of studies performed and consequently the overall number of animals in in-vivo studies. Short transcriptomic dose analysis studies (~ 5 days) have been shown to be a reliable alternative for the estimation of chronic toxicities for environmental chemicals^22,23^. This has not yet been achieved for drugs, which require a more thorough risk assessment than chemicals. Several efforts have been exerted to analyze the optimum durations needed for discovering chronic toxicities in drug development.

Upon the analysis of 59 sub-chronic and chronic studies, Idrizbegovic et al. concluded that most toxicities were identified in studies up to 3 months duration﻿ and that compound termination in the clinical phase due to new findings in chronic toxicity studies were rare (about 10%)^24^. Roberts et al. compared the incidence and severity of target organ toxicities observed in first time in man (FTiM) (≤ 6 weeks) studies versus sub-chronic/chronic (≥ 3 months) studies for 39 candidate drugs^25^. A balance between the appearance of new target organ toxicities (n = 31) and the resolution of existing target toxicities (n = 29) was observed, thus challenging the assumption that toxicity is aggravated by long-term exposure to a candidate drug.

Other publications comparing toxicity end points of short and long-term studies, were limited to large molecules^26,27^.

In this work, we explore the likelihood of identifying new adverse events with longer durations of studies through a statistical approach. The importance of reproducibility of statistical approaches used in preclinical research was highlighted by Lazic and coworkers and was therefore considered in this study^28^.

We hereby present an open-source R workflow, termed CSL-Tox, which facilitates the comparison between acute and chronic toxicity studies, with respect to the adverse findings reported. In this study, a large cohort of in vivo toxicological rodent and non-rodent studies were analyzed (a total of 192 in-house and in-house sponsored CROs studies) with representation across both small and large molecules (25 and 18 molecules respectively). Throughout this comparison, we explore the sufficiency of short-term studies in detecting adverse findings and the corresponding NOAELs. Bayesian statistics and likelihood ratios, which are commonly used methods in non-clinical and clinical concordance studies, were adopted in this comparison^29–31^. The open-source R program is available for application to other similar datasets. This can help researchers in analyzing and comparing additional short-term and long-term studies and therefore contribute to the minimization of study durations.

Finally, we discuss challenges faced during this study such as cumbersome data extraction and annotation of treatment related findings and current efforts to overcome these challenges.

## Methods

### Workflow

**Figure 2:**
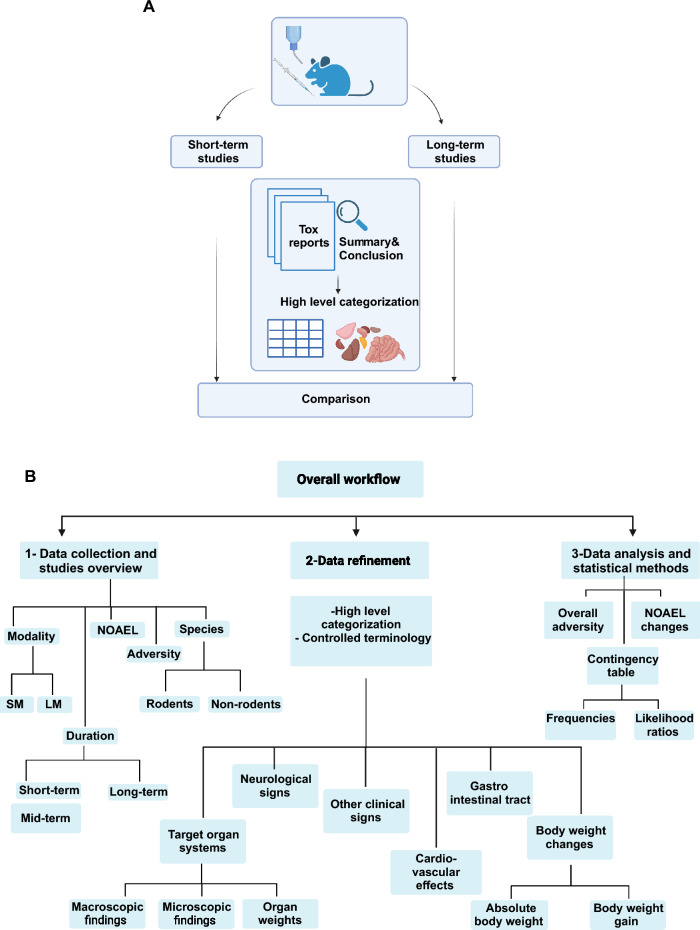
(**A**) A graphical summary of the “CSL-Tox” workflow. (**B**) An overview of the steps performed in this work and implemented in the “CSL-Tox” workflow.

An overall view of the work is given in Fig. 2. Figure 2A represents a graphical summary of the CSL-Tox workflow which can be summarized in comparison of high-level categorized adverse findings extracted from the “Summary” and “Conclusion” sections of toxicology reports for the short-term and long-term studies. More details on the high-level categorization of the findings is given in Table 1. The overview of the work is explained in more details in Fig. 2B and can be divided into three main steps as follows: (1) data collection and studies overview (modality, species, duration); (2) data refinement (including the high-level categorization of the findings, converting the finding terms into the controlled terminology, and flagging the treatment related findings); (3) Data analysis of the results (calculation of the overall adversity, NOAEL changes and the Bayesian contingency tables).

**Table 1:** Summary of the 9 adverse events categories further aggregated into 6 high level categories.

Categories	High level categories	Event examples
Absolute body weight	Body weight changes	Increase, decrease
Body weight gain		Increase, decrease
Macroscopic findings	Post-mortem findings (target organ system)	Decrease in size (thymus gland)
Microscopic findings		Hepatocyte hypertrophy (liver)
Organ weights		Decrease (liver)
Cardiovascular effects	Cardiovascular effects	Increased heart rate, QTc prolongation
Neurological clinical signs	Neurological clinical signs	Hypoactivity, tremors
GIT clinical signs	GIT clinical signs	Salivation, vomit
Other clinical signs	Other clinical signs	Irregular respiration

Examples of events are provided for each category. Full details on the events are provided in Tables S1 and S2.

### Data collection and overview of the studies

In-house study report databases were searched for general toxicity studies conducted on rodent and non-rodent species. Both modalities, small and large molecules were included. No restriction was placed on the timeframe for the studies collected (the studies retrieved ranged from 1999 to 2020). However, for a molecule to be considered in the dataset, short or/and mid and long-term studies should have been conducted and the molecule have progressed to First In Human (FIH) studies. Therefore, the main inclusion criteria of the molecules in the dataset were based on the study duration:

#### Short-term and midterm studies

A general toxicity study with a duration of less than 20 weeks should have been conducted. We further distinguish between two classes of studies: general toxicity studies with a duration of up to a maximum of 6 weeks (short-term) and studies with a duration of more than 6 weeks and less than 20 weeks (midterm).

#### Long-term studies

A general toxicity study with a duration of at least 26 weeks should have been conducted (long-term). According to the current guidelines, those studies are required to support continued clinical development and marketing of molecules, hence they are conducted at the latest stage of the drug development process. As a direct consequence, molecules that were terminated in initial stages due to adversity or other commercial reasons were excluded from the database. Furthermore, this requirement resulted in the exclusion of anticancer pharmaceuticals that were developed after 2010, when the revised ICHS9 guideline restricting the maximum duration of a nonclinical study for advanced cancer indications to 13 weeks, came into effect^15^.

Information on the study and the therapeutic molecules was gathered from the study reports such as the study duration, species, modality of the molecule, route of administration, dose interval, no observed adverse effect level and adversity or treatment-related findings were registered in the database. The latter two can be described as follows:

#### No observed adverse effect level (NOAEL)

NOAEL is defined as the highest dose administered at which no adverse effects are observed^32^. The NOAEL can be a representative measure of how the toxicity, associated with a specific treatment, progresses as the duration of the study increases and was therefore considered in this work. For an overall analysis of the effect of the study duration on the NOAEL, short-term and midterm studies for each species have been grouped together and the lowest specified NOAEL has been identified per molecule. This was used as a basis to assess the influence of long-term treatment on the NOAEL. If more than one long-term study was conducted for a specific species, the lowest of the defined NOAELs was used in the comparison.

#### Adverse treatment-related findings

“Treatment related” findings are defined as a finding that is considered related to the drug. “Adverse” is a term indicating "harm" to the test animal, while non-adverse indicates lack of harm and is usually attributed to the expected pharmacology of the test item. The “Summary” and “Conclusion” section of the study reports were the main source for the extraction of toxic effects that were considered treatment-related. Detailed information on treatment-related findings (categories specified below) recorded in the toxicity study was collected. Specifically, the dose at which the effect appeared, and most importantly whether it was considered adverse by the toxicologist in charge. The level of severity and reversibility and any extra information related to the effect or its cause (if stated, e.g. whether it was considered secondary to stress) were also recorded.

The findings were grouped into 9 broad categories. The categories, as well as examples of effects/events belonging to each of the categories are shown in Table 1. Full details on the events included in each category (except for microscopic findings) are provided in the supplementary material (Table S1A–E). The detailed microscopic findings are also provided in the supplementary material (Table S2). For the effects belonging to the categories “organ weights”, “macroscopic findings” and “microscopic findings” their target organs were also recorded. Information on toxicokinetics was not included as it was considered out of scope for this particular analysis.

### Data refinement (high level effect aggregation and controlled terminology)

Firstly, species were grouped into rodents (rat and mouse) and non-rodents (cynomolgus monkey, minipig, marmoset and dog). Secondly doses were normalized for each study i.e. since dosing intervals may vary between studies, doses were computed per day in order to be able to compare different studies). Finally, since many of the toxicity effects showed low prevalence, grouping of the effects was performed in order to gain a better understanding of the overall incidence of adverse events observed in long-term, midterm and short-term studies. No grouping was performed for the four categories*:* neurological clinical signs, other clinical signs, gastro-intestinal tract, and cardiovascular effects. The findings within each category are presented in detail in Table S1A–D respectively. On the other hand, the three categories macroscopic findings, microscopic findings and organ weights were grouped into “post-mortem findings” and the two remaining categories body weight and body weight gain, were grouped into “body weight changes” as follows:

#### Post-mortem findings (target-organ system)

The “macroscopic findings”, “microscopic findings” and “organ weights” categories were merged into one and the effects were associated with the target organ system affected. For example, all the postmortem effects belonging to the mentioned categories and affecting organs in the endocrine system were grouped under the high-level effect “endocrine system”. The target systems grouping was performed according to Table S2A, where it is described in detail which organs or tissues constitute each target system. The macroscopic findings are described in Table S1E and the microscopic findings are described separately in Table S2B due to the considerable amount of information.

#### Body weight changes

“Body weight” and “body weight gain” categories were combined into one category named “body weight changes” since both reflect the influence of the therapeutic molecule on the weight of the animals.

A summary of the presence/absence of the findings within the described categories was included in the dataset.

#### Overall adversity measure

Based on the previous categories, the “overall adversity” of each compound was registered in the dataset, where it was labelled as “No” if zero adverse effect was observed and “Yes” if at least one adverse effect was observed.

### Data analysis and statistical methods

The statistical methods implemented in this study and used for the analysis of the adverse events were adopted from the work of Clark et al.^29^. We used Bayesian statistics with a 2 by 2 contingency table to measure the concordance between observations made in the initial short and midterm general toxicity studies and the long-term toxicity studies. We treat the observations made in the short and mid-term toxicity studies as a diagnostic test for observations made in the long-term toxicity studies and use the statistical methods developed for the evaluation of the efficacy of these diagnostic tests. The contingency tables were calculated after the high-level grouping of adverse events (explained previously) was performed and was done across the two categories rodents and non-rodents.

**Figure 3:**
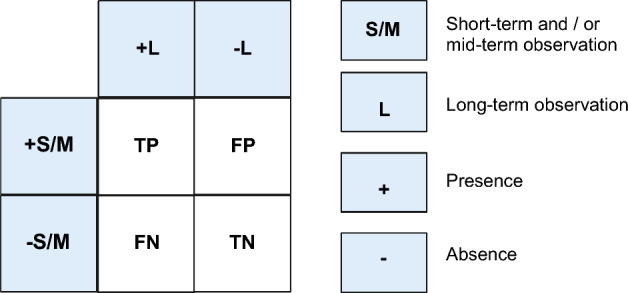
Contingency table used in the statistical analysis of the findings.

The values in the contingency table, which represent number of molecules in each of the four categories for a specific high-level effect are explained in Fig. 3 and were generated as follows:

- *True positives* Count of molecules for which the high-level effect was observed in the long-term study as well as in the short-term or/and mid-term study.
- *False positives* Count of molecules for which the high-level effect was observed in the short-term or/and mid-term study but not in the long-term study
- *True negatives* Count of molecules for which the high-level effect was not observed in any study (neither short/mid nor long-term)
- *False negatives* Count of molecules for which the high-level effect was not observed in the short-term or/and mid-term study but was observed in the long-term study

#### Likelihood ratios

Likelihood ratios were used to determine the statistical connection between the toxicity observations made in the initial toxicity studies and the long-term study. Likelihood ratios were considered the appropriate metric since they combine the knowledge of both the sensitivity and the specificity of the model^21^. Likelihood ratios also have the advantage of being independent from the prevalence, allowing for the comparison of high-level effects with different prevalence in the dataset.

The positive likelihood ratio (LR+) is defined as sensitivity/(1 − specificity) whereas the inverse Negative Likelihood (iLR−) is defined as specificity/(1 − sensitivity). Likelihood ratios were calculated according to Eqs. (1) and (2) respectively. The p-values for the relationships in the 2 by 2 contingency tables were computed using Fisher’s exact test and the interpretation of likelihood ratios were adopted from Chien and Khan’s work and is listed in Table 2^33^. Only statistically significant likelihood ratios (p-value < 0.05) were considered in the analysis and a cut-off of 5 was drawn to indicate a “high” positive or inverse negative likelihood ratio.

**Table 2:** Interpretation of likelihood ratios.

+ LR and − iLR	Significance
> 10	Large and often conclusive shifts in probability
5–10	Moderate shifts in probability
2–5	Small, but sometimes important, shifts in probability
1–2	Alters probability to a small, and rarely important, degree

1 $$+LR= \frac{TP \times (FP+TN)}{FP \times (TP+FN)}$$

2 $$-iLR- = \frac{FN \times \left(FP+TN\right)}{TN \times \left(TP+FN\right)}$$

#### Frequency or percentage of false positives and false negatives

Miss-predictions of findings suggested by the short-term studies were estimated by the percentage/frequency of false positives [the events that were observed in short-term studies and not observed in long-term studies, calculated by Eq. (3)] and the percentage or frequency of false negative [the events that were observed in long-term studies and missed by the short-term studies, calculated by Eq. (4)].

3 $$\%FN= \frac{FN}{FN+TP+TN+FP}\times 100$$

4 $$\%FP= \frac{FP}{FN+TP+TN+FP}\times 100$$

### Implementation and tools

**Figure 4:**
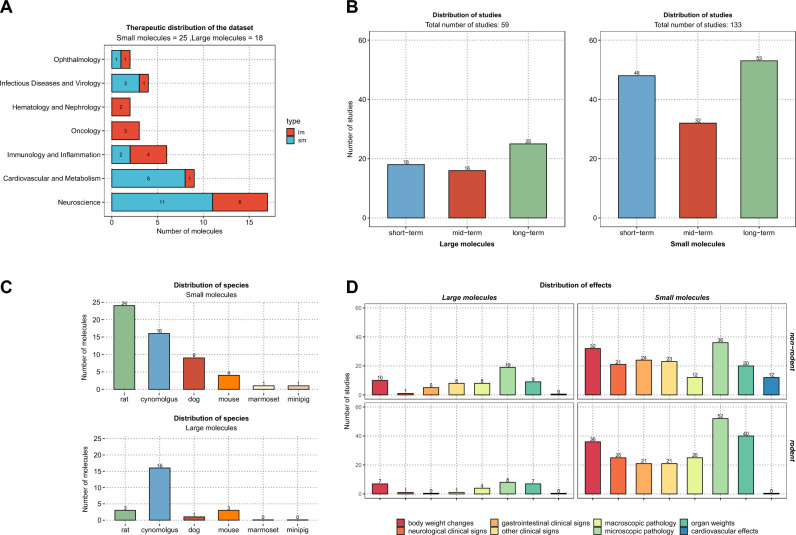
An overall illustration of the compiled dataset with respect to therapeutic areas covered, study durations, species tested and adverse events observed. (**A**) A bar plot showing therapeutic areas covered by the dataset, the number of molecules belonging to each therapeutic area is indicated on the corresponding bar. Each bar is colored according to the type of molecule. (**B**) A bar plot showing the number of studies with respect to the duration (short, mid, and long term). The number of studies present in each duration category is indicated on the top of the corresponding bars. (**C**) A bar plot showing the number of compounds tested per species (for rodents and non-rodents). (**D**) A bar plot showing the number of studies for which adverse events were registered in rodent and non-rodent species (number of studies is indicated on the top of each bar). The bars are colored according to the adverse event categories.

**Figure 5:**
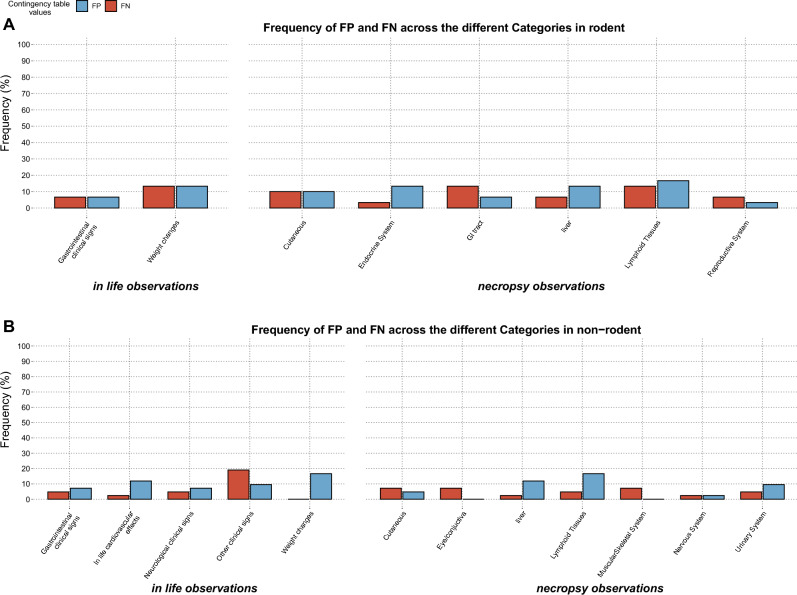
Frequency or percentage of false positives and false negatives of the findings across the different categories in (**A**) Rodents, (**B**) Non-rodents.

All the data manipulation and statistical analysis was implemented in R studio (version 3.5.1)^34^. Figures 1 and 2 were created using www.biorender.com and Package ggplot (version 3.3.2) was used for plotting Figs. 3, 4 and 5. The CSL-Tox code and tutorial are available at the GitHub repository: https://github.com/Roche/CSL-Tox.

The functions corresponding to the steps explained in the workflow and implemented in the software are provided in Table 3.

**Table 3:** Key functions available in the workflow.

Category	Functions	Description
Data exploration	TherapeuticAreas DifferentSpecies StudiesPerFindingCat	Plots an overview of the therapeutic areas, species, and findings available in the dataset
Data refinement	ReadingData	Reads data from a .txt file and put it in the required format
	ChangeData	Applies controlled terminology (Tables S1, S2) to the 9 categories of findings and groups them into the 6 high level categories explained in Table 1
Data analysis	AdversitySummary	Calculates the overall adversity per modality (small molecules and large molecules)
	NOAELChange	Calculates NOAEL increase, decrease or same from short and mid to long-term studies. The lowest NOAEL doses are compared in case of multiple studies
	LikelihoodRatio	Calculates contingency table, + LR, − iLR and corresponding Fisher test p-values per species (rodents or non-rodents)

## Results

### Overview of the dataset

A representation of the compiled dataset is given in Fig. 4 with respect to the therapeutic areas covered by the compounds, the study durations, the type, and number of species for each study and the overall distribution of adverse events for rodents and non-rodents.

A total of 43 molecules met the inclusion criteria, including 25 small molecules and 18 large molecules (composed of 16 conventional monoclonal antibodies(mAbs), 2 bispecific mABs (Compound-R&J) and 2 recombinant mABs (Compound-D&C). A wide range of therapeutic areas is represented as shown in Fig. 4A, with “Neurosciences” being the most represented area. The full dataset is provided in the supplementary material (Tables S3, S4).

The distribution of the studies across the different specified duration classes is shown in Fig. 4B. For the large molecules, a total number of 61 general toxicity studies are included in the dataset (25 long-term studies). As for small molecules, a total number of 133 general toxicity studies are included in the dataset (53 long-term studies). A short-term and a mid-term study were not always conducted as part of a new molecule’s toxicity testing, with the short-term study being preferred to the mid-term study.

In Fig. 4C, the bar plots show the number and type of species used in the toxicity studies for both small and large molecules.

As seen in the figure, the rat was the predominant rodent species (24/25) while cynomolgus (16/25) followed by the dog (9/25) were the main non-rodent species.

With regards to large molecules, the current guideline for large molecules, ICH S6(R1), recommends the testing of novel medicinal products in pharmacologically relevant species only; if possible, a rodent and non-rodent. Frequently the only such species is the cynomolgus monkey, due to the higher sequence homology between the human and cynomolgus monkey proteome.

Cynomolgus monkey was the predominant selected non-rodent species in large molecules (16/18). One molecule was only tested in the dog and one molecule was only tested in the mouse. The rat was the selected rodent species for 3 large molecules, whereas the mouse was selected for the remaining 2 molecules.

Figure 4D shows the distribution of all the findings recorded within the studies in the specified categories. The frequency of findings observed for large molecules was lower than that for small molecules, which can be attributed to the higher target specificity of large molecules and thus lower probability of off-target effects. “Microscopic findings” followed by “organ weights” and “body weight changes” were the categories with the most frequently observed effects in both small molecules and large molecules.

### Overall adversity

As explained in the “Methods” section, an overall adversity measure was assigned to gain an overall understanding on the concordance of findings between the short/mid-term studies and the long-term studies. The overall adversity observations are displayed in Table 4 for large molecules, where a concordance between short/mid-term and long-term studies is observed for the majority of the molecules (17 out of 18). 13 out of the 17 molecules that showed concordance did not show any adverse events in neither the short/mid nor the long-term studies. Four out of 18 molecules showed adverse effects in both the short/mid and the long-term studies. For all 4 compounds, adverse findings were already apparent latest in the 13-w studies and was usually missed by the 2-w studies. For example adverse erythropoietic effects in Compound-O was already seen in the 8-w studies and confirmed in the 26-w non-rodent studies and missed by the 2-w study. Increase in Red Blood Cells (RBC) volume was seen in Compound-P in the 4-w and 13-w rodent studies. For Compound-Q, pathological findings were seen in all rodent study durations (4-w, 13-w, 26-w) while none was seen in non-rodents.Se vere ocular inflammation was seen in both the 13-w and 26-w non-rodent studies for Compound-R. These results might indicate that a duration of maximum 13-w (~ 3 months) could be sufficient to understand the safety profile of mABs. For one out of the 18 large molecules (Compound-E) an antibiotic resistant infection, resulting in a skin lesion, was observed in only one animal in the long-term studies and not in the short-term study. The short-term study is not considered fully unpredictive in this case since it the skin infection was most likely not compound related but could not be ruled out. Large molecules therefore showed an overall high concordance in the findings between short/mid and long-term studies.

**Table 4:** A comparison between the occurrence of adverse effects in large molecules for the short and mid-term studies versus long-term studies per compound.

Large molecule	Short or mid-term study	Long-term study
Compound-A to compound-D	No adverse effects observed	
Compound F to compound-N		
Compound-E	No adverse effects observed	Adverse effects observed
Compound-O to compound-R	Adverse effects observed	

For small molecules, the short/mid-term toxicity studies were able to predict the adverse effects in long-term studies for the majority of the molecules (18 out of 25) as seen in Table 5. A difference in findings between the short/mid- and long-term studies was seen for 6 out of 25 small molecules. For only 2 of those (Compound-6 and Compound-7), new adverse effects were reported only in the long-term studies. For Compound-6 (4-w, 12-w, and 39-w), similar findings were observed in the 4-w and 12-w study (thyroid and parathyroid findings in 4-w study were reversible and appeared in 12-w study). However, in the 39-w study other adverse effects were observed, such as decreased prostate weights. Animals were generally younger in age at study start in long-term studies and effects on the prostate were only observed as animals matured. Short term studies in older animals may have missed this. For Compound-7 [4 w, 26 w] axonal degeneration was observed in the long-term study and was minimal. Other findings such as food consumption changes were predicted by the short-term studies.

**Table 5:** A comparison between the occurrence of adverse effects in small molecules for the short and mid-term studies versus long-term studies per compound.

	Short or mid-term study	Long-term study
Compound-1 to compound-5	No adverse effects observed	
Compound-6 and compound-7	No adverse effects observed	Adverse effects observed
Compound-8 to compound 11	Adverse effects observed	No adverse effects observed
Compound 12 to compound 25	Adverse effects observed	

Upon the analysis of dose levels administered for Compounds-8 to 11 in the performed studies, dose level adjustments (decrease in doses from short-term to long-term studies) were observed for Compound-9 and 11 which might be the reason of the disappearance of adverse events in the long-term studies while no dose level adjustments were observed for Compound-8 and 10. Detailed information can be found in the supplementary material (Table S5A).

### Progression of the “no observed adverse effect levels” (NOAEL) with study duration

As previously mentioned, the determination of a NOAEL is a very important objective of preclinical toxicity studies^2^. Since toxicity may progress with longer duration of exposure (with respect to incidence and severity), we were interested in understanding NOAEL changes from short/mid-term to long-term studies. As seen in Table 6, for small molecules, a NOAEL was identified for rodent studies in 23 molecules; where 8 decreased, 12 stayed the same and 3 increased from short/mid-term to long-term studies. In non-rodent studies a NOAEL was identified for 22 molecules; where 10 decreased, 7 stayed the same, 5 increased.

**Table 6:** NOAEL changes for small and large molecules in rodent and non-rodent studies with a focus on NOAEL decrease attributed to progression of adverse effects.

N = number of molecules^a^	Small molecules (SM)			Large molecules (LM)			
	Same	Increase	Decrease	Same	Increase	Decrease	
Rodents *N*_*SM-rodents*_ = *23* *N*_*LM-rodents*_ = *6*	12	3	8 (ProgNoael^b^ = 4/8)	4	0	2 (ProgNoael^b^ = 2/2)	
	ProgNoael_SM-rodents_ = 4/23			ProgNoael_LM-rodents_ = 2/6			ProgNoael_rodents_ = 6/29
Non-rodent *N*_*SM-nonrodents*_ = *22* *N*_*LM-nonrodents*_ = *17*	7	5	10 (ProgNoael^b^ = 4/10)	8	6	3 (ProgNoael^b^ = 1/3)	
	ProgNoael_SM-nonrodents_ = 4/22			ProgNoael_LM-nonrodents_ = 1/17			ProgNoael_non-rodent_ = 6/39

^a^The numbers indicated represent the total number of molecules for which the NOAEL increased/decreased/stayed the same for rodent and non-rodent studies in small molecules (N_SM-rodents_, N_SM-nonrodents_) and large molecules (N_LM-rodents_, N_LM-nonrodents_) respectively. For some molecules both rodent and non-rodent studies were performed.

^b^Decrease in NOAEL due to progression of adverse effects.

For large molecules, a NOAEL was identified in for 6 large molecules and decreased for 2 molecules, and it stayed the same for 4 molecules from short-term to long-term studies.

In rodent studies a NOAEL was identified for 17 molecules and the NOAEL stayed the same or increased for most of the molecules (stayed the same for 8 molecules, increased for 6 molecules) and decreased for 3 molecules.

For both, small and large molecules, the NOAEL increase was attributed to higher doses tested in longer-term studies. The NOAEL decreased in the longer-term studies, due to progression of findings for 4 out of 23 small molecules and 2 out of 6 large molecules in rodent studies. In non-rodent studies, the NOAEL decrease was attributed to progression of adverse effects for only 1 out 17 large molecules and 4 out of 22 small molecules. Otherwise, the NOAEL decrease was due to lower doses tested in the longer duration studies.

In conclusion, the overall NOAEL decrease due to progression of adverse events was seen for only 20% (6 out of 29) and 15.3% (6 out of 39) of the molecules in rodent and non-rodent studies respectively. It is worth to say that for some compounds these findings had already been observed in the shorter duration studies but were not considered adverse due to low incidence or severity.

As explained in the “Methods” section, the NOAEL change algorithm compares the NOAEL raw values of the short and mid vs long-term studies. The results included in Tables 4, 5 and 6 are the NOAEL changes after the toxicologists’ revisions of the algorithm outcome and interpretation of the study reports. Few discrepancies were observed between the algorithm outcome and the toxicologists’ interpretations in the NOAEL changes [provided in the supplementary material (Table S5B)]. For example, algorithm expected an increase in the NOAEL Compound K in rodents and non-rodents while the toxicologist interpreted the NOAEL as staying the same. The “errors” identified by the toxicologists in the NOAEL algorithm changes were attributed to:

1. Changes in some studies were irrelevant to human, therefore the toxicologist disregarded these studies in the NOAEL change estimation.
2. Changes were due to differences in the study design and not due to better/worse tolerability in long term studies.
3. Difference in the definition of adversity of a molecule from one toxicologist to another (due to better acquaintance with the molecule for example). Since adversity is driven by findings that have functional consequences on organs, increased knowledge of presence or absence of functional consequences may also change adversity definitions from a pathologist’s perspective.

### Positive and negative likelihood ratios

Positive and negative likelihood ratios of both adverse and non-adverse findings are given in Tables 7, 8, 9 and 10 providing an overview on the ability of short-term studies to predict the appearance or non-appearance of adverse findings (or non-adverse findings) in the long-term studies. For example, a high positive likelihood ratio indicates that there is a high probability to observe adverse events in long-term studies, given that they appear in the short-term studies. A high inverse negative likelihood ratio indicates that there is a high probability of not observing the adverse events in long-term studies if they were not observed in the short-term studies. Therefore, the high positive or inverse likelihood ratios demonstrate the concordance of findings between short-term and long-term studies. A high + LR (> 5) was seen for the majority of findings for non-rodents (Table 7) except for weight changes, other clinical signs and lymphoid tissue findings which showed a lower + LR (~ 4). More importantly, very high likelihood ratios were observed for all the adverse findings in non-rodents (Table 8).

**Table 7:** Values of contingency tables along with the statistically significant positive and negative inverse likelihood ratios of the non-adverse findings in non-rodents.

Findings	TP	FP	FN	TN	+ LR	− iLR	p_value*	Species
Eye/conjunctiva	2	0	3	37	Inf*	1.67	0.01	Non-rodents
Muscular skeletal system	2	0	3	37	Inf	1.67	0.01	
Nervous system	2	1	1	38	26	2.92	0.01	
cutaneous	10	2	3	27	11.15	4.03	0	
Neurological clinical signs	6	3	2	31	8.5	3.65	0	
Gastrointestinal clinical signs	10	3	2	27	8.33	5.4	0	
liver	7	5	1	29	5.95	6.82	0	
In life cardiovascular effects	3	5	1	33	5.7	3.47	0.02	
Urinary system	3	4	2	33	5.55	2.23	0.03	
Weight changes	14	7	0	21	4	Inf	0	
Lymphoid tissues	8	7	2	25	3.66	3.91	0	
Other clinical signs	7	4	8	23	3.15	1.6	0.03	

*Inf Infinity. Infinity values were obtained due to the presence of zero false positives.

*p values for Fisher’s exact test.

**Table 8:** Values of contingency tables along with the statistically significant positive and negative inverse likelihood ratios of the adverse findings in non-rodents.

Adverse findings	TP	FP	FN	TN	+ LR	− iLR	p_value	Species
Nervous system	2	0	0	40	Inf	Inf	0	Non-rodents
Lymphoid tissues	2	1	0	39	40	Inf	0	
liver	2	1	1	38	26	2.92	0.01	
Neurological clinical signs	3	2	2	35	11.1	2.36	0.01	
Weight changes	2	5	0	35	8	Inf	0.02

**Table 9:** Values of contingency tables along with the statistically significant positive and negative inverse likelihood ratios of the non-adverse findings in rodents.

Findings	TP	FP	FN	TN	+ LR	− iLR	p_value	Species
Reproductive system	4	1	2	23	16	2.87	0	Rodents
Gastrointestinal clinical signs	7	2	2	19	8.17	4.07	0	
GI tract	5	2	4	19	5.83	2.04	0.01	
Cutaneous	5	3	3	19	4.58	2.3	0.02	
Endocrine system	13	4	1	12	3.71	10.5	0	
Liver	14	4	2	10	3.06	5.71	0	
Lymphoid tissues	9	5	4	12	2.35	2.29	0.04	
Weight changes	14	4	4	8	2.33	3	0.02

**Table 10:** Values of contingency tables along with the statistically significant positive and negative inverse likelihood ratios of the adverse findings in rodents.

Adverse findings	TP	FP	FN	TN	+ LR	− iLR	p_value	Species
Liver	3	0	1	26	Inf	4	0	Rodents
Lymphoid tissues	2	2	1	25	9	2.78	0.04	

Less concordance was observed on the level of the -iLR of the non-rodent findings, where low values were observed for the Eye/conjunctiva, Muscular skeletal system, Nervous system, neurological clinical signs, In-life cardiovascular effects, urinary system, lymphoid tissues, and other clinical signs. A very high -iLR (equal to infinity) was observed for all the non-rodent adverse findings except for the liver and neurological clinical signs where low -iLR were observed (2.92 and 2.36 respectively).

Lower + LR were observed for the majority of rodent findings (Table 9), except for the reproductive system, gastro-intestinal clinical signs, GIT, and cutaneous findings. The same trend was observed for the − iLR. Regarding the adverse findings in rodents (Table 10) high + LR values were observed for all the adverse findings while lower − iLR values were observed for the liver and lymphoid tissues (− iLR < 5).

### Frequency of false positives and false negatives

**Figure 6:**
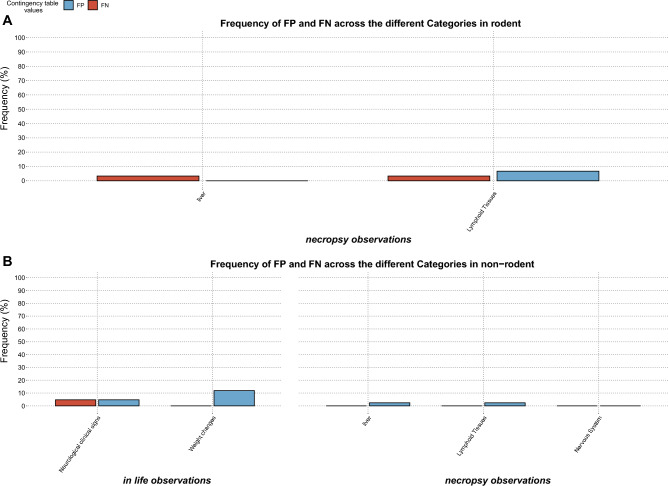
Frequency or percentage of false positives and false negatives of the **adverse** findings across the different categories in (**A**) Rodents, (**B**) Non-rodents.

Percentages of false positives and false negatives predicted by the short-term studies are represented for all the findings (Fig. 5) and adverse findings (Fig. 6). Similar to the likelihood ratios, only statistically significant contingency tables were analyzed, and frequencies were calculated as explained in the “Methods” section. As seen in Fig. 4, frequencies of false positives and false negatives for findings in both rodents (Fig. 5A) and non-rodents (Fig. 5B) are all below 20%, denoting that the short-term studies successfully predicted the presence (True positives) or absence (True negatives) of the findings by 80%. Categories with false negative frequencies higher than 10% were: GI tract, lymphoid tissues, and weight changes for rodent, while for non-rodents only the other clinical signs category showed a false negative frequency higher than 10%. Categories with false positive frequencies higher than 10% were weight changes, endocrine system, liver and lymphoid tissues for rodents and In-life cardiovascular effects, weight changes, liver, and lymphoid tissues for non-rodents.

In Fig. 6 frequencies of false positives and false negatives for the adverse findings are displayed for rodents (Fig. 6A) and non-rodents (Fig. 6B), all adverse findings showed frequencies less than 10% except for false positive weight changes in non-rodents.

Observations drawn from the false positive and false negative frequencies are in accordance with the likelihood ratios results where the overall results showed that the majority of the adverse findings exhibited high + LR and − iLR (except for lymphoid tissues adverse findings in rodent where − iLR = 2.78). However, it is of importance to analyze both measures since they do not quantify miss-predictions similarly. The cut-off of “High” or “Low” likelihood ratios shows an impact on the results. For example, considering the adverse findings in rodents, low values of false positives and false negative frequency (6.6% and 3.3% respectively) were observed for the lymphoid tissue, indicating a good predictability of the short-term studies for the long-term adverse events. These frequencies might not be a sufficient indicator for the prediction capability of the short-term studies, since upon investigation of the likelihood ratios we observe that despite a high + LR being observed for both findings (according to our chosen likelihood ratio cut-off), a low − iLR was (2.78) was seen. This indicates that there is a high probability to observe these adverse events in long-term studies, even if they were not observed by the short-term studies. A similar case can be observed upon the analysis of adverse findings in rodents, where liver and neurological clinical signs showed low false positive and false negative frequencies (2.3%) and (4.7%) respectively, while they exhibited low -iLR (2.92 and 2.36 respectively). Both false negatives and low -iLR are considered concerning in this analysis, since the false negative indicates the presence of adverse events in the long-term studies and their absence in the short-term studies, and a low -iLR indicates that if an adverse event was not seen in short-term studies, there is still a good probability that it will be seen in the long-term study.

## Discussion

Minimization of the number of in-vivo studies is a challenging goal and requires the analysis of a vast amount of in-vivo data for high confidence conclusions to be made.

Currently, mandatory disclosure of data and code associated with animal toxicity research publications is quite rare, leading to a difficulty in the reproducibility of the studies and the corresponding statistical analysis^35^. This work emphasizes on the importance of reproducibility in the analytical tools used in animal toxicity studies and overcoming the obstacle of data confidentiality.

In this study, toxicity endpoints were extracted from 192 toxicity studies associated with 18 large and 25 small molecules. A generally good concordance was seen between short-term and long-term studies on the overall adversity level, where a good predictivity was observed for the adverse events between short and long-term studies in most of the cases, with only 1 out of 18 large molecules (Compound-E) and 2 out of 25 small molecules (Compound-6 and 7) being unpredictive.

For the four large molecules (Compound-O, P, Q & R) where adverse events were observed in both short and long-term studies, 13-w study durations seemed to be sufficient to reveal the observed adverse events. These observations (previously described in the “Results” Section) coincides with the work of Chien et al., where the authors deduced that very few novel adverse findings are revealed in long-term toxicity studies for mABs and that a 3-month study is usually sufficient to support late stage clinical development (for 142 mABs, 6-month studies uncovered adverse findings for only 13% of the mABs while short-term studies (≤ 3-months) was sufficient to reveal adverse events for 71% of the mABs)^36^. These results can promote the reduction of study durations for large molecules.

The number of molecules, studies and adverse events extracted in our work however seemed to be insufficient to generate a decisive score or coefficient that can determine whether a long-term study could be skipped, especially for small molecules.

The presence of robust and sufficient datasets seems to be a recurrent need to provide clear evidence that can convince authorities to alter current guidelines concerned with study durations, number of animals, number of species and optimizing other pillars of toxicity testing without presenting a risk to human safety. For example, Prior et al. explored the opportunities for the use of only one species in long-term toxicity studies through the analysis of toxicity studies for 172 candidate drugs (92 small molecules and 80 large molecules). The authors showed that for most large molecules (e.g. antibodies), only one relevant non-rodent species was usually used and was sufficient to explore the toxicities of the molecules, which coincides with the ICHS6(R1) guidelines^14^. However, they highlighted the need for more robust data to enable the extrapolation of this recommendation to small molecules and other biologics where two species are currently proposed (e.g. therapeutic proteins and peptides), without defining the exact number of studies required to reach a clear recommendation.

It is indeed difficult to give an accurate estimation of “sufficient and robust” data in terms of number of molecules and studies required to reach a firm conclusion (in our case to omit long-term studies). However we expect that such estimation can be achieved through continuous dialogue with the authorities.

Nevertheless, the workflow adopted in the presented analysis is available as an open-source tool for other researchers to reproduce and adapt. The dataset and the detailed code for the statistical methods used (contingency tables, likelihood ratios and frequency of false positives and negatives) are provided to allow other scientist to reassess the analysis methodology and propose other methods that might fit better this study.

This ensures the statistical reproducibility of the work, facilitates the expansion of this analysis and its extrapolation to other datasets which might help in concretely addressing our main question, whether it is necessary to perform long-term studies in toxicity testing.

The open-source availability of our dataset can also help address other prominent challenges in animal testing, such as that presented by Prior et al.^21^.

Inclusion of additional information on candidate drugs (especially small molecules) such as the main biological target, off-target profile, mechanism of action and if possible, the chemical structure of the compound could be valuable for building read-across predictive tools and should be considered in future work.

In addition to the concordance in overall adversity between short and long-term studies, other interesting observations were made. Firstly, the same reversible adverse events were observed for Compound-6 in both the short-term (4 w) and mid-term (12-w) studies, while other adverse events were revealed in the long-term 39 w study. This gives rise to the question whether mid-term studies could be skipped and thus considering a concordance analysis between short-term and mid-term findings in future studies might be of importance.

Secondly, the analysis of the reasons behind the changes in the NOAEL was beneficial and might have a potential impact for decision making. For example, the NOAEL increase from short-term to long-term studies for the majority of the molecules was due to higher doses tested in the long-term studies. Finally, analyzing both likelihood ratios and false positive/false negative frequencies was of importance due to the different significance given by each measure. It is also important to analyze the contingency table and determine which measure fits best to the setting of the study, for example in our case it was important to analyze both the false negatives and false positives and not to limit the analysis to the true positives or true negatives. Disregarding a false positive compound might result in deprioritizing a compound for the wrong reasons, while disregarding a false negative would lead to missing adverse events risks upon skipping longer studies. Therefore, both measures constituted equal importance in this study.

Multiple challenges were faced during this work, mainly being associated with the time-consuming nature of manual data extraction and its’ refinement. While in-vivo study reports are a valuable source of information on the safety findings of a drug candidate, they are usually provided in PDF format, which was the case for our studies. Therefore, manual extraction was required in addition to expertise in assessing the annotation of the findings as “treatment related” or “non-treatment related”. Attempting novel technologies for data extraction such as natural language processing and text mining techniques can enhance the cumbersome data extraction process, especially for the extraction of certain key findings such as study duration, dose, species and NOAEL^37^.

The importance of reproducibility is highlighted through the challenges faced in the cumbersome extraction of adverse events and NOAELs from the reports and highlights the necessity of the standardization of the report format to facilitate the application of analytical tools in preclinical toxicity studies. Other similar challenges were encountered in this work, such as the verification of the controlled terminology of the findings, categorization of the effects and their attribution to the appropriate target organ system. A more systematic data extraction process would be needed for a large-scale application of the work.

Since the FDA has recently put in place a recommendation for a standard format for non-clinical data regulatory submission, known as “The Standard Exchange of Non-clinical Data” (SEND), this should facilitate the analysis of preclinical data in the future. Other efforts have been exerted in the standardization of data (with respect to pathology terms), such as the International Harmonization of Nomenclature and Diagnostic Criteria for Lesions in Rats and Mice (INHAND)^38^. Cross-institutional consortia and collaborations have also been exerted to address many of the previously mentioned challenges. For example, the eTOX project which highlighted the importance of legacy data sharing and leveraging the valuable in-vivo toxicity data existing in pdf formats within the archives of large pharmaceutical companies^39^. The e-TOX project was succeeded by the translational safety assessment consortium (eTRANSAFE), which covered many aspects of preclinical and clinical toxicity such as the translatability of in-vivo toxicities of a new chemical entity (NCE) into the clinical dimension and the extent or relevance of these findings to man, the application of text mining and other techniques for extraction, visualization and analysis of data from preclinical and clinical reports and prediction of safety events through computational tools^40^. Confidentiality and sensitivity of the in-vivo data is another challenge faced for large-scale application of such studies and was tackled by both the eTOX and eTRANSAFE projects This data has not been incorporated in our analysis due to the absence of sufficient in-house molecules in the e-tox database with both short-term and long-term studies.

In this study our assessment of the ability of short-term studies to predict long-term effects of small and large molecules was limited to the comparison of findings from both durations.

However, correlation and comparison of the short-term and long-term findings with the clinical adverse findings could also be a way, not only to minimize long-term studies, but to minimize in-vivo studies in general. Analysis of the clinical data corresponding to these molecules was not within the scope of this study and therefore should be considered in future work.

Adopting innovative approaches for the prediction and extrapolation of time dependent findings like time series analysis models could also be considered. However expanding the dataset and analyzing more studies using the developed workflow would be needed prior to the application of such predictive approaches and highlights again the importance of reproducibility of analytical tools used in preclinical toxicity.

## Conclusion

Shortening the preclinical development pipeline and minimization of in-vivo studies are long sought goals in the drug discovery and development field. In this work, an in-house dataset was built through manual extraction of data from toxicity study reports. We performed a statistical comparison between short-term and long-term toxicity endpoints to explore whether short-term studies are enough to detect adverse effects exhibited by small and large molecules. The methodology applied in this work was compiled into an open-source software, freely available for other users. The software implements important functions that facilitate this comparison, such as an overview of the distribution of the effects exhibited in the studies, study durations and the species used in the analysis, calculation of the overall adversity of the molecules, the NOAEL changes of molecules and the likelihood ratios of the adverse findings. Although our analysis showed promising results, the major question, whether it is possible to omit long-term studies remains insufficiently addressed, due to the relatively small dataset size and associated adverse effects employed in this analysis. A larger number of molecules and expansion of the current dataset is required to reach a tangible conclusion that could potentially impact the toxicologists’ decisions on minimizing the study durations and number of studies performed. We encourage other researchers to implement, ameliorate this workflow and extend its application to public and in-house datasets.

### Supplementary Information

Supplementary Tables.

## Data Availability

The CSL-Tox workflow is freely available for users and can be found at https://github.com/Roche/CSL-Tox. A detailed R markdown tutorial explaining the steps of the workflow is provided. The data necessary to reproduce this work is deposited in the same GitHub repository (Tables S3 and S4).

## Abbreviations

CSL-Tox: Comparison of short-term and long-term toxicity studies
NCE: New chemical entity
ICH: The international council for harmonization of technical requirements for pharmaceuticals for human use
3Rs: Replacement, reduction and refinement
SP: Safety pharmacology
FIH: First in human
ADRs: Adverse drug reactions
SM: Small molecules
LM: Large molecules
NOAEL: No observed adverse effect level
GLP: Good laboratory practice
ST: Short term
MT: Mid term
LT: Long term
+ LR: Positive likelihood ratio
− iLR: Inverse negative likelihood ratio
