# Supplementary material for: CSL-Tox: an open-source analytical framework for the comparison of short-term and long-term toxicity end points and assessing the need of chronic studies in drug development
Source: Sci Rep. 2023 Sep 8;13:14865. doi: 10.1038/s41598-023-41899-4 (PMC10491674; doi:10.1038/s41598-023-41899-4)
Supplement: Supplementary file 1 — Supplementary Tables. [file 41598_2023_41899_MOESM1_ESM.docx]

**Table S1.** Detailed summary on all the findings extracted from the reports included under the high-level categories previously defined in Table 1.

| **Used Terminologies** | **Preferred Terminilogy** |
| --- | --- |
| **(A) GIT clinical signs** | GIT clinical signs |
|  |  |
| vomitus |  |
| emesis |  |
| oral discharge |  |
| fecal abnormalities |  |
| reduced feces |  |
| soft feces |  |
| unformed feces |  |
| white discoloration of feces |  |
| diarrhea |  |
| liquid feces |  |
| excessive salivation |  |
| salivation |  |
| swollen abdomen |  |
| bedding in mouth |  |
| mouth rubbing |  |
| **(B) Neurological clinical signs** | Tremors/convulsions |
| localised tremor |  |
| tremor |  |
| twitching |  |
| increased seizure risk |  |
| convulsive behavior |  |
| clonic tonic convulsions with tremor |  |
| convulsive episodes |  |
| slow movements | Hyperactivity/hypoactivity |
| hypoactivity |  |
| reduced activity |  |
| decreased activity |  |
| reduction in locomotor activity |  |
| sedated behavior |  |
| subdued behavior |  |
| lethargy |  |
| apathy |  |
| reduced reaction to outside stimulus |  |
| sedating effect |  |
| unresposiveness |  |
| increased activity |  |
| agitation with atypical behavior |  |
| retlessness |  |
| rolling gait | abnormal gait/posture |
| stiff gait |  |
| abnormal gait |  |
| bent gait |  |
| hunched gait |  |
| impaired mobility |  |
| abnormal mobility |  |
| unsteady gait |  |
| staggering gait |  |
| staggered movement |  |
| ataxia |  |
| staggering ataxia |  |
| limited usage of left hindlimb |  |
| modified coordination |  |
| incoordination |  |
| paddling |  |
| hunched posture |  |
| abnormal posture |  |
| stiff posture |  |
| hunched back |  |
| prominent backbone |  |
| distorted body |  |
| lying position |  |
| recumbent posture |  |
| lateral posture |  |
| prone |  |
| prone posture |  |
| prostration |  |
| recumbency |  |
| tail elevation |  |
| hunched position |  |
| head movement | abnormal behavior |
| pushing their head through bedding |  |
| side to side movement of the head |  |
| yawning |  |
| abnormal vocalisation |  |
| whimpering |  |
| vocalisation |  |
| vocalising |  |
| piloerection | piloerection |
| **(C)Other clinical signs** |  |
| panting respiration | irregular respiration |
| irregular respiration |  |
| deep breathing |  |
| irregular breathing |  |
| noisy breathing |  |
| panting |  |
| labored respiration |  |
| discolored haircoat of the perioral region | abnormal haircoat |
| rough haircoat |  |
| ruffled fur |  |
| fur loss |  |
| hairloss |  |
| half closed eyes | partially closed or closed eyes |
| partially closed eyes |  |
| semi closed eyes |  |
| closed eyes |  |
|  |  |
| poor condition | Morbidity |
| debilitation |  |
| morbidity |  |
| thin body |  |
| thin appearance |  |
| emaciation |  |
| pale |  |
| paleness |  |
| moribund condition |  |
|  |  |
| enterobacter cloacae infection in the skin | skin infection |
|  |  |
| skin thickening | skin thickening |
|  |  |
| flaky skin | skin lesions |
| skin lesions |  |
| skin scales |  |
| scabby skin |  |
| focal skin lesions |  |
| soiled areas |  |
| multifocal skin lesions |  |
| redenning of the extremities |  |
| erythema of the ears and extremities |  |
| wet lesion |  |
| scabbing |  |
| scabs |  |
| red/black skin |  |
| skin discoloration | Skin discoloration |
| black skin at eyelids |  |
| black skin at periorbital areas |  |
| swelling at the face | Face edema |
| swelling at eyelids |  |
| swelling at periorbital areas |  |
| swelling |  |
| subcutaneous edema |  |
| nasal discharge | discharge |
| eyes discharge |  |
| lacrymation |  |
| lacrymation and secretion |  |
| cold extremities | hypothermia |
| cold to touch |  |
| coldness to the touch |  |
| decreased body surface temperature |  |
| hypothermia |  |
| decreased body temperature |  |
| sunken eyeballs | dehydration |
| dehydration |  |
| dehydration weakness |  |
| depression of ERG amplitudes | ERG changes |
| ERG scotopic B wave amplitudes were depressed |  |
| ocular inflammation | Ocular findings |
| perivascular sheathing |  |
| hazy media |  |
| vitreous cells |  |
| limbal corneal pigmentation |  |
| white particles in the aqueous humor |  |
| brown particles in the aqueous humor |  |
| white particles in the vitreous body |  |
| reduced IOP |  |
| increased retinal nerve fiber layer thickness | OCT changes |
| **(D)Vital Signs- Cardiovascular effects** |  |
| increased heart rate | cardiovascular effects |
| decreased QT intervals |  |
| RR interval decrease |  |
| reduced heart rate |  |
| bradycardia |  |
| QTc prolongation |  |
| increase in QTcV interval |  |
| PR prolongation |  |
| second degree atrioventricular block |  |
| extrasystoles |  |
| **(E)Macroscopic pathology** |  |
|  |  |
| increase | increase |
| decrease | decrease |
| small |  |
| firm | abnormal shape/surface |
| inflated |  |
| raised area |  |
| abnormal shape |  |
| rounded |  |
| granulated surface with medullary rays |  |
| rough surface |  |
| soft |  |
|  |  |
| swollen | edema |
| edema |  |
| cortical hemorrhage (adrenal gland) | hemorrhage |
| retinal hemorrhage |  |
| subretinal hemorrhage |  |
| hemorrhage |  |
| pallor | discoloration |
| pale foci |  |
| pale focus |  |
| pale |  |
| cortical pigmentation (adrenal gland) |  |
| mottling |  |
| mottled |  |
| dark |  |
| red discolourations |  |
| red |  |
| increase in grey and white and tan foc |  |
| clay-like discoloration |  |
| discolored |  |
| discolored |  |
| tan or white discoloration |  |
| grey and white discoloration |  |
| reddening of the glandular mucosa |  |
| reddish colored foci in the glandular region |  |
| thickened subcutaneous tissue and nodules | skin thickening, skin lesions |
| thickened subcutaneous tissue and scabs |  |
| thickened subcutaneous tissue and nodules and hematomas and scabs |  |
| thick sites |  |
| sores |  |
| lesions |  |
| thickened |  |
| skin thickening |  |
| injection site mass |  |
| scales |  |
|  |  |
| ascites | effusions |
| hydropericardium |  |
| hydrothorax |  |
| distension | distension |
| calculus | calculus |
| erosions | erosions |
| decrease activity | decreased activity |
| increased adipose tissue (it refers again to neck, face, skin) | increased adipose tissue |
| prolonged persistence of injuries | prolonged persistence of injuries |
| dental abnormalities | dental abnormalities |
| tarsal hyperemia | tarsus bone hyperemia |
| vasculitis | vasculitis |
| decreased sensory conduction velocity |  |
| decreased motor conduction velocity |  |

**Table S2**.(A) Details on target organ constitute the high level target systems and **(B)** The microscopic findings with respect to each target organ.

| **Organ system** | **Organs/Tissues** |
| --- | --- |
| Nervous System | “nerve”,”tibial", "nerve,trigeminal", "ganglion,dorsal root", "hypoglossal nerve", "glossopharyngeal nerve", "brain", "nerve,peroneal","nerve,sural", "spinal cord,cervical", "spinal cord,thoracic", "ganglion,cervical", "sciatic nerve", "spinal cord", "central nervous system","peroneal nerve", "sural nerve","choroid plexus","hypothalamus", "nervous system" |
| GI tract | "stomach", "gastrointestinal tract", "jejunum", "ileum", "esophagus", "colon", "tooth",  "hard palate", "oral cavity", "tongue", "gland,brunner's", "gallbladder","abdomen","gall bladder","mesentery","large intestine","small intestine", "cecum", "duodenum","rectum","abdomen","retroperitoneum", "gitract" |
| Reproductive system | “prostate gland", "testis", "vagina", "male reproductive system", "ovary", "uterus",  "epididymis","female reproductive system","sex organ", "preputial gland", "clitoral gland", "seminal vesicle","reproductive system" |
| MuscularSkeletal_System | “muscle,skeletal", "bone", "sternum", "shoulder", "axilla","tail","tarsus bone","axillary","flank",  "neck","skeletal muscle", "ankle", "knee","hindlimb", "femur joint","stifle joint", "thigh muscle", "knee joint","femur", "muscularskeletal system" |
| Endocrine_System | "thyroid gland", "adrenal gland", "parathyroid gland", "pancreas", "pituitary gland","endocrine system" |
| Exocrine_System | "mammary gland", "salivary gland,sublingual", "gland,lacrimal", "parotid gland", "salivary gland","submandibular gland", "gland,harderian", "prostate gland", "exocrine system" |
| Respiratory_system | "lung", "nasal turbinate", "larynx","pleural cavities", "trachea","respiratory system” |
|  |  |
| Cutaneous | “skin","injection site", "adipose tissue, brown","mesenteric adipose tissue","most tissues","brown adipose tissue","cutaneous" |
| Eye_conjuctiva | "eye", "conjunctiva", "eye/conjuctiva" |
| Urinary_system | "bladder", "urinary system", "ureter", "kidney","urinary system" |
| Lymphoid_Tissues | "axillary lymph node", "tonsil", "submandibular lymph node", "lymphoid tissues", "lymph node", "mesenteric lymph node", "spleen","bone marrow", "thymus gland", "mandibular lymph node","inguinal lymph node","lymphoid tissues" |
| Cardiovascular_System | "heart","injection vein", "aorta","cardiovascular system" |
| Whole_Body | “terminal body","whole body" |

**(A)**

**(B)**

| **Terminology** | **Controlled Terminology** | **Higher Level Grouping** |  |
| --- | --- | --- | --- |
| **kidney** |  |  |  |
| tubular basophilic granules | tubular degeneration/regeneration |  |  |
| basophilic granules | tubular degeneration/regeneration |  |  |
| increased hyaline droplets | tubular hyaline droplets, increased |  | brown seperate |
| degeneration/regeneration | tubular degeneration/regeneration |  |  |
| tubular foamy/granular macrophages | tubular foamy/granular macrophages |  |  |
| foamy macrophages | foamy/granular macrophages |  |  |
| basophilic granules in proximal tubular epithelial cells | tubular degeneration/regeneration |  |  |
| degeneration/regeneration of proximal tubular cells, | tubular degeneration/regeneration | inflammation/infiltrate | inflammation |
| pelvic inflammation | pelvic inflammation |  | infiltrate |
| transitional cell hyperplasia in the renal pelvis | pelvic transitional cell hyperplasia |  |  |
| mononuclear cell infiltrate in the pelvic/peripelvic area | pelvic infiltrate |  |  |
| pyelonephritis | pyelonephritis | hypertrophy/hyperplasia | hyperkeratosis |
| pyelitis | pyelitis |  | hypertrophy |
| tubular vacuolation | tubular vacuolation |  | hyperplasia |
| increased levels of pigment | increased pigmentation |  |  |
| increased incidence of the chronic progressive nephropathy | chronic progressive nephropathy |  | atrophy |
| extramedullary hematopoiesis | extramedullary hematopoiesis |  |  |
| tubular dilatation | tubular dilatation |  |  |
| epithelial attenuation and regeneration | epithelial attenuation, epithelial regeneration |  |  |
| inflammatory cell infiltration | inflammation, infiltrate |  | congestion |
| proteinaceaous casts | Cast (tubular) |  | hemorrhage |
| tubular basophilia | Basophilic tubule (tubular degeneration/regeneration) |  | edema |
| nephropathy | chronic progressive nephropathy |  |  |
| tubular regeneration | Basophilic tubule (tubular degeneration/regeneration) | degeneration | degeneration/regeneration |
| degeneration and fibrosis | tubular degeneration/regeneration |  | single cell necrosis/ apoptrosis |
| increased incidence of tubular basophilia | Basophilic tubule (tubular degeneration/regeneration) |  |  |
| increased incidence of hyaline droplets | tubular hyaline droplets, increased | adipocyte changes | adipocyte accumulation |
| tubular hyaline droplets | increased tubular hyaline droplets |  |  |
| glomerular vacuolation | glomerulopathy |  |  |
| glomerulopathy | glomerulopathy |  |  |
| adipocytes | adipocyte accumulation |  |  |
| urothelial lesions | pelvic lesions |  |  |
| hypertrophy of the lining epithelium of collecting ducts | epithelial degeneration/regeneration |  |  |
| tubular degeneration | tubular degeneration/regeneration |  |  |
| atrophy | atrophy |  |  |
| vacuolation | vacuolation |  |  |
| dilation | dilation |  |  |
| infiltrates of vacuolated macrophages | foamy/granular macrophages |  |  |
| intranuclear inclusions | intranuclear inclusions |  |  |
| glomerulonephritis | glomerulonephritis |  |  |
| tubular nephropathy | chronic progressive nephropathy |  |  |
| cortical atrophy | cortical atrophy |  |  |
| tubular atrophy with interstitial fibrosis | tubular atrophy, fibrosis |  |  |
| mixed cell infiltrates | infiltrate |  |  |
| granuloma formation | Granuloma |  |  |
| pelvic dilatation | pelvic dilatation |  |  |
| transitional cell hyperplasia | pelvic transitional cell hyperplasia |  |  |
| perirenal inflammation | perirenal inflammation |  |  |
| granular casts | cast (tubular) |  |  |
| inflammation | inflammation |  |  |
| granulomas | granuloma |  |  |
| vascular congestion | Congestion |  |  |
| congestion | Congestion |  |  |
| increase in the severity of hemosiderosis | Hemosiderin |  |  |
|  |  |  |  |
|  |  |  |  |
|  |  |  |  |
| **lymph node** |  |  |  |
| basophilic granules | lymphoid degeneration/regeneration |  |  |
| increased sinus macrophages | macrophages |  |  |
| increased lymphoid cellularity | hyperplasia |  |  |
| vacuolated macrophages | foamy/granular macrophages |  |  |
| lymphoid depletion | lymphoid depletion |  |  |
| edema | edema |  |  |
| plasmocytosis | plasmocytosis |  |  |
| lymphocyte depletion | lymphoid depletion |  |  |
| necrosis | necrosis |  |  |
| lymphoid stimulation | lymphoid stimulation |  |  |
| Sinus histiocytosis | Sinus histiocytosis |  |  |
|  |  |  |  |
| **liver** |  |  |  |
| granular kupfer cells | granular kupfer cells |  |  |
| hepatocellular single cell necrosis/apoptosis | hepatocyte apoptosis/single cell necrosis |  |  |
| hepatocyte basophilic granules | granular hepatocytes |  |  |
| basophilic granular kupffer cells | granular kupfer cells |  |  |
| kupffer cell hypertrophy | kupffer cell hypertrophy |  |  |
| hepatocyte hypertrophy | hepatocyte hypertrophy |  |  |
| increased mononuclear cell infiltrates | infiltrate |  |  |
| centrilobular hypertrophy | hepatocyte hypertrophy |  |  |
| centrilobular hepatocyte hypertrophy | hepatocyte hypertrophy |  |  |
| periportal hepatocellular vacuolation | hepatocyte vacuolation |  |  |
| increased mitoses | increased mitosis/increased mitotis figures |  |  |
| kupffer cell pigmentation | kupffer cell pigmentation |  |  |
| diffuse increase in lipid droplets | lipid accumulation |  |  |
| increased hepatocyte vacuolation | hepatocyte vacuolation |  |  |
| lipid accumulation | lipid accumulation |  |  |
| prussian blue positive pigment in kupffer cells | hemosiderin |  |  |
| increased incidence and severity of hepatocyte vacuolation | hepatocyte vacuolation |  |  |
| diffuse and centrilobular/midzonal hepatocellular lipid deposits | lipid accumulation |  |  |
| multinucleate hepatocytes | multinucleated hepatocytes |  |  |
| hepatocellular pigmentation | hepatocyte pigmentation |  |  |
| bile duct hyperplasia | bile duct hyperplasia |  |  |
| clear cell foci | foci |  |  |
| basophilic tigroid foci | foci |  |  |
| biliary hyperplasia | bile duct hyperplasia |  |  |
| portal inflammation | portal inflammation |  |  |
| yellow and brown pigments in kupffer cells | kupffer cell pigmentation |  |  |
| macrophages | macrophages |  |  |
| kupffer cell vacuolation | kupffer cell vacuolation |  |  |
| periacinar hypertrophy | hepatocyte hypertrophy |  |  |
| diffuse hypertropy | hypertrophy |  |  |
| periacinar vacuolation | hepatocyte vacuolation |  |  |
| periacinar lipid deposits | lipid accumulation |  |  |
| increase in the severity of hemosiderosis | hemosiderin |  |  |
| kupffer cell hemosiderin granules | Hemosiderin |  |  |
| diffuse hepatocellular lipid deposits, | lipid accumulation |  |  |
| centrilobular hepatocellular lipid deposits, | lipid accumulation |  |  |
| midzonal hepatocellular lipid deposits | lipid accumulation |  |  |
| shift of lipid deposits from periportal to periacinar or midzonal | lipid accumulation |  |  |
| increased frequency and severity of hepatocytic glycogen vacuolation | hepatocyte glycogen vacuolation |  |  |
| increased severity of pas staining for glycogen storage | hepatocyte glycogen vacuolation |  |  |
| hypocellularity of hematopoietic tissue | decreased hematopoiesis |  |  |
| increased hematopoiesis | increased hematopoiesis |  |  |
| basophilic granules in kupffer cells, | granular kupffer cells |  |  |
| eosinophilic intranuclear inclusions in hepatocytes | intranulcear inclusions |  |  |
| higher levels of glycogen vacuolation, | hepatocyte glycogen vacuolation |  |  |
| lower hepatic lipid levels, | lipid depletion |  |  |
| higher hepatocellular glycogen levels | hepatocyte glycogen accumulation |  |  |
| inflammatory cell foci | inflammation |  |  |
| glycogen depletion | hepatocyte glycogen depletion |  |  |
| decreased oil red-o fat staining | hepatocyte lipid depletion |  |  |
| multinucleated hepatocytes | multinucleated hepatocytes |  |  |
| tigroid basophilic foci | foci |  |  |
| cellular alteration | foci |  |  |
| hepatocellular adenoma | hepatocyte adenoma |  |  |
| increased extramedullary hematopoesis | extramedullary hematopoiesis |  |  |
| reduced glycogen content in hepatocytes | hepatocyte glycogen depletion |  |  |
| multifocal cytoplasmic granularity of hepatocytes | hepatocyte granularity |  |  |
| diffuse ito cell hyperplasia | ito cell hyperplasia |  |  |
| vascular congestion | congestion |  |  |
| extramedullary erythropoiesis | extramedullary hematopoiesis |  |  |
| hepatic glycogen vacuolation | hepatocyte glycogen vacuolation |  |  |
| kupffer cell hemosiderin granules | Hemosiderin |  |  |
|  |  |  |  |
| **Injection site** |  |  |  |
| basophilic granules | basophilic granules |  |  |
| granular macrophages | foamy/granular macrophages |  |  |
| granulomatous inflammation | inflammation |  |  |
| subcutaneous fibrosis | fibrosis |  |  |
| collagen degradation | collagen degradation |  |  |
| subcutis necrosis | necrosis |  |  |
| edema | edema |  |  |
| hemorrhage | hemorrhage |  |  |
| mononuclear cell response | infiltrate |  |  |
| acute inflammation | inflammation |  |  |
| inflammatory cell infiltration | inflammation,infiltrate |  |  |
| increased perivascular inflammation | inflammation |  |  |
| collagen deposition | fibrosis |  |  |
| acanthosis | acanthosis |  |  |
| inflammatory lesions | inflammation |  |  |
| incidence and severity of inflammatory cell foci | inflammation |  |  |
| hyperkeratosis | hyperkeratosis |  |  |
| epidermal hyperplasia | epidermal hyperplasia |  |  |
| increased mononuclear, perivascular inflammatory cell foci | inflammation |  |  |
| inflammation | inflammation |  |  |
| subcutis inflammation | inflammation |  |  |
| subcutis adipose tissue atrophy | adipose tissue atrophy | Blood findings | hematopoiesis |
| mononuclear infiltrate | infiltrate |  |  |
| lymphoplasmacytic infiltrates | infiltrate |  |  |
| mixed infiltrate | infiltrate |  |  |
| hypertrophy of the muscle fiber in the skin and subcutis | hypertophy |  |  |
| perivascular infiltrate of mononuclear cells in the subcutis | infiltrate |  |  |
| perivascular infiltrate of mononuclear cells in the dermis | dermal infiltrate |  |  |
| serocellular crust (*2- would be nice to be clustered with something) | serocellular crust |  |  |
|  |  |  |  |
| **lung** |  |  |  |
| equivocal low grade vascular changes |  |  |  |
| increase in the incidence and grade of foamy macrophages | foamy/granular macrophages |  |  |
| increased infiltrate of intra-alveolar macrophages | macrophages |  |  |
| increased incidence of alveolar foamy macrophages | foamy/granular macrophages |  |  |
| congestion | congestion |  |  |
|  |  |  |  |
| **testis** |  |  |  |
| tubular degeneration/atrophy | tubular degeneration/atrophy |  |  |
| granular macrophages | foamy/granular macrophages |  |  |
| increase of degenerated spermatocytes | spermatocyte degeneration |  |  |
| tubular degeneration | tubular degeneration/atrophy |  |  |
| degeneration of seminiferous tubules | tubular degeneration/atrophy |  |  |
| tubular vacuolation | tubular vacuolation |  |  |
| degeneration of germ cells | germ cell degeneration |  |  |
| multinucleate cells | multinucleate cells |  |  |
|  |  |  |  |
| **eye** |  |  |  |
| retinal degeneration | retinal degeneration |  |  |
| multifocal degeneration of the retina | retinal degeneration |  |  |
| vacuolation of ganglion cell layer and inner nuclear layer and outer plexiform layer | retinal vacuolation |  |  |
| keratopathy | keratopathy |  |  |
| mixed cell infiltrate in the inner retina and optic disc and vitreous | infiltrate |  |  |
| perivascular plasma cell in the optic disc | infiltrate |  |  |
| mild-optic disc perivascular plasma cell | infiltrate |  |  |
| anterior uvea plasma cell | infiltrate |  |  |
| anterior uvea mononuclear infiltrate | infiltrate |  |  |
| limbus plasma cell | infiltrate |  |  |
| limbus mononuclear infiltrate | infiltrate |  |  |
| inner retina degeneration | retinal degeneration |  |  |
| inner retina pigmented macrophages | infiltrate? |  |  |
|  |  |  |  |
| **skin** |  |  |  |
| epidermal hyperplasia | epidermal hyperplasia |  |  |
| epidermal single cell necrosis | epidermal single cell necrosis |  |  |
| inflammation | inflammation |  |  |
| erosion/ulcer | erosion/ulcer |  |  |
| serocellular crusts | serocellular crust |  |  |
| hyperkeratosis | hyperkeratosis |  |  |
| mixed inflammatory infiltrate | infiltrate, inflammation |  |  |
| apoptotic/dyskeratotic keratinocytes | abnormal keratinocytes |  |  |
| epidermal hyperplasia/hyperkeratosis | epidermal hyperplasia, epidermal hyperkeratosis |  |  |
| parakeratosis | abnormal keratinocytes |  |  |
| necrosis of the superficial layers of epidermis, | epidermal necrosis |  |  |
| epidermal and dermal inflammation | epidermal inflammation,dermal inflammation |  |  |
| focal, often ulcerative, dermatitis | ulcerative dermatitis |  |  |
| subcutaneous inflammation | subcutaneous inflammation |  |  |
| adipocyte accumulation | adipocyte accumulation |  |  |
| edema | edema |  |  |
| subcutis fat atrophy | subcutaneous adipose tissue atrophy |  |  |
| multifocal superficial ulcerative dermatitis | ulcerative dermatitis |  |  |
| subcutis-atrophy of the adipose tissue | subcutaneous adipose tissue atrophy |  |  |
| hypertrophy of the cutaneous muscle | cutaneous hypertrophy |  |  |
| epidermal necrosis | epidermal necrosis |  |  |
| dermal necrosis | dermal necrosis |  |  |
| single cell keratinocyte necrosis | single cell necrosis |  |  |
|  |  |  |  |
| **thymus gland** |  |  |  |
| atrophy | atrophy |  |  |
| decreased lymphoid cellularity | atrophy |  |  |
| increased lymphoid cellularity | hyperplasia |  |  |
| decreased lymphocytes | atrophy |  |  |
| decreased cortical lymphocytes | atrophy |  |  |
| lymphoid atrophy | atrophy |  |  |
| increased lymphocytolysis | increased lymphocytolysis |  |  |
| delayed involution | delayed involution |  |  |
| diffuse atrophy | atrophy |  |  |
| lymphocyte depletion | lymphoid depletion |  |  |
| necrosis | necrosis |  |  |
| vascular congestion | congestion |  |  |
| thymic involution | involution |  |  |
|  |  |  |  |
| **pancreas** |  |  |  |
| single cell necrosis | single cell necrosis |  |  |
| enlarged adipocytes | enlarged adipocytes |  |  |
| adipocyte accumulation | adipocyte accumulation |  |  |
| zymogen depletion | zymogen depletion |  |  |
| increased occurrence of apoptotic acinar cells | exocrine  cell apoptosis |  |  |
| exocrine epithelial single cell necrosis | exocrine epithelial single cell necrosis |  |  |
|  |  |  |  |
| **colon** |  |  |  |
| distension | distension |  |  |
| single cell necrosis | single cell necrosis |  |  |
|  |  |  |  |
| **epididymis** |  |  |  |
| decreased sperm | decreased sperm |  |  |
| ductular epithelium degeneration/necrosis | degeneration/necrosis |  |  |
|  |  |  |  |
| **female reproductive system+male reproductive system** |  |  |  |
| estrous cycle arrest | estrous cycle arrest |  |  |
| atrophy | atrophy |  |  |
|  |  |  |  |
| **Gastrointestinal tract** |  |  |  |
| crypt epithelial single cell necrosis | mucosa epithelial apoptosis/single cell necrosis |  |  |
| degeneration/necrosis | degeneration/necrosis |  |  |
| apoptosis/single cell necrosis | apoptosis/single cell necrosis |  |  |
| dilation | dilation |  |  |
| mucosa edema | mucosa edema |  |  |
| submucosa edema | submucosa edema |  |  |
| crypt apoptosis | mucosa apoptosis |  |  |
|  |  |  |  |
| **bone marrow** |  |  |  |
| decreased cellularity | hypocellularity |  |  |
| increase micronucleated erythrocytes | increased hematopoiesis |  |  |
| increased lymphoid cellularity | hyperplasia |  |  |
| lower incidence of fat in the sternum | adipose tissue atrophy |  |  |
| lower incidence of fat in the femur | adipose tissue atrophy |  |  |
| hypercellularity | hyperplasia |  |  |
| hyperplasia in the sternum | hyperplasia |  |  |
| fat reduction | adipose tissue atrophy |  |  |
| intracellular and extracellular perl’s stained iron | hemosiderin |  |  |
| myeloid hyperplasia | hyperplasia |  |  |
| adipocyte accumulation | adipocyte accumulation |  |  |
| hypocellularity of hematopoietic tissue | hypocellularity |  |  |
| enlarged adipocytes | enlarged adipocytes |  |  |
| absence of erythroid precursors in sternum | decreased hematopoiesis |  |  |
| atrophy | hypocellularity |  |  |
| atrophy in the femoral adipose tissue | adipose tissue atrophy |  |  |
| increased hematopoiesis | increased hematopoiesis |  |  |
| increased production of nucleated erythrocytes | increased hematopoiesis |  |  |
|  |  |  |  |
| **tooth** |  |  |  |
| odontoblast degeneration/necrosis | degeneration/necrosis |  |  |
| dental dysplasia | dysplasia |  |  |
|  |  |  |  |
| **hard palate** |  |  |  |
| **epithelial single cell necrosis** | epithelial single cell necrosis |  |  |
|  |  |  |  |
| **mammary gland** |  |  |  |
| atrophy | atrophy |  |  |
| increased intracellular brown granular pigment | increased pigmentation, granularity |  |  |
| diffuse lobuloalveolar hyperplasia | hyperplasia |  |  |
| acinar hyperplasia | hyperplasia |  |  |
| enlarged adipocytes | enlarged adipocytes |  |  |
| adipocyte accumulation | adipocyte accumulation |  |  |
|  |  |  |  |
| **thyroid gland (+ parathyroid gland)** |  |  |  |
| follicular cell hypertrophy | follicular hypertrophy |  |  |
| follicular cell hyperplasia | follicular hyperplasia |  |  |
| diffuse follicular cell hypertrophy | follicular hypertrophy |  |  |
| follicular distension | follicular distension |  |  |
| increased incidence and severity of flattened thyroid gland follicular cell epithelium | follicular dilatation |  |  |
| adipocyte accumulation | adipocyte accumulation |  |  |
| follicular dilatation | follicular dilatation |  |  |
| follicular cell adenoma | follicular adenoma |  |  |
|  |  |  |  |
| **lymphoid tissues** |  |  |  |
| increased lymphoid cellularity | hyperplasia |  |  |
| decreased lymphocytes | lymphoid depletion |  |  |
|  |  |  |  |
| **pituitary gland** |  |  |  |
| mononuclear cell infiltrates | infiltrate |  |  |
| hypertrophy of cells of the adenohypophysis | hypertrophy |  |  |
| increased vacuolation | vacuolation |  |  |
| increased incidence of vacuolation | vacuolation |  |  |
| hypertrophy | hypertrophy |  |  |
| increased incidence of hypertrophy | hypertrophy |  |  |
| pars distalis hypertrophy | pars distalis hypertrophy |  |  |
| single cell hypertrophy | hypertrophy |  |  |
|  |  |  |  |
| **sciatic nerve+ nerve,tibial+nerve,trigeminal +ganglion,dorsal root+ hypoglossal nerve+glossopharyngeal nerve** |  |  |  |
| mononuclear cell inflammation | infiltrate,inflammation |  |  |
| axonal degeneration | axonal degeneration |  |  |
| perineurium degeneration | perineurium degeneration |  |  |
| mononuclear infiltrate | infiltrate |  |  |
| axonal degeneration in the dorsal funiculus | axonal degeneration |  |  |
| vacuolation of neurons | neuron vacuolation |  |  |
| choroid plexus infiltrate | infiltrate |  |  |
|  |  |  |  |
| **urinary system** |  |  |  |
| mononuclear cell infiltration | infiltrate |  |  |
|  |  |  |  |
| **spleen** |  |  |  |
| increased lymphoid cellularity | hyperplasia |  |  |
| decreased lymphocytes | lymphoid depletion |  |  |
| increase in haemopoiesis | increased hematopoiesis |  |  |
| decreased germinal center and marginal zone cellularity | lymphoid depletion |  |  |
| vacuolated macrophages | foamy/granular macrophages |  |  |
| extramedullary hematopoiesis | extramedullary hematopoiesis |  |  |
| hemosiderosis | hemosiderin |  |  |
| increased pigment (iron) | hemosiderin |  |  |
| increased compensatory hemopoiesis | extramedullary hematopoiesis |  |  |
| increased hematopoiesis | increased hematopoiesis |  |  |
| infiltrates of vacuolated macrophages in the red pulp | foamy/granular macrophages |  |  |
| lower incidence of hematopoiesis | decreased hematopoiesis |  |  |
| decreased hematopoiesis | decreased hematopoiesis |  |  |
| necrosis | necrosis |  |  |
| vascular congestion | congestion |  |  |
| increased production of nucleated erythrocytes | increased hematopoiesis |  |  |
| congestion | congestion |  |  |
|  |  |  |  |
| **tonsil** |  |  |  |
| lymphoid depletion | lymphoid depletion |  |  |
| increased lymphoid cellularity | hyperplasia |  |  |
|  |  |  |  |
| **larynx** |  |  |  |
| epithelial hyperplasia | epithelial hyperplasia |  |  |
| parakeratosis | abnormal keratinocytes |  |  |
| apoptotic keratinocytes | abnormal keratinocytes |  |  |
| inflammation | inflammation |  |  |
| increased mitosis | increased mitosis (or mitotic figures) |  |  |
| apoptosis | apoptosis/single cell necrosis |  |  |
| single cell necrosis | apoptosis/single cell necrosis |  |  |
| ulceration and necrosis of the vocal fold epithelium | epithelial ulcer, epithelial necrosis |  |  |
| reactive squamous hyperplasia | epithelial hyperplasia |  |  |
| respiratory epithelium regeneration | epithelial regeneration |  |  |
| epithelial degeneration | epithelial degeneration |  |  |
|  |  |  |  |
| **ovary** |  |  |  |
| increased corpora lutea | increased corpora lutea |  |  |
| increased vacuolation | vacuolation |  |  |
| adipocyte accumulation | adipocyte accumulation |  |  |
|  |  |  |  |
| **uterus** |  |  |  |
| vacuolation | vacuolation |  |  |
| vacuolated cells | vacuolation |  |  |
|  |  |  |  |
| **jejunum+ileum+esophagus+conjuctiva** |  |  |  |
| increased apoptosis, single cell necrosis | apoptosis/single cell necrosis |  |  |
| distension | distension |  |  |
| increased mitosis | increased mitosis (or mitotic figures) |  |  |
| inflammation | inflammation |  |  |
| single cell necrosis (mucosal epithelium) | mucosa epithelial single cell necrosis |  |  |
| mixed infiltrates | infiltrate |  |  |
|  |  |  |  |
| **adrenal gland** |  |  |  |
| increased cortical microvacuolation (zona fasciculata but occasionally in the zona glomerulosa) | cortical vacuolation |  |  |
| lipid depletion | lipid depletion |  |  |
| increased cytoplasmic eosinophilia | lipid depletion |  |  |
| diffuse cortical hypertrophy | cortical hypertrophy |  |  |
| diffuse cortical vacuolation | cortical vacuolation |  |  |
| Diffuse hypertrophy of cortical zona reticularis | cortical hypertrophy |  |  |
| cortical atrophy | cortical atrophy |  |  |
| cortical hypertrophy | cortical hypertrophy |  |  |
| hypertrophy of the zona glomerulosa | cortical hypertrophy |  |  |
| cortical foamy alveolar macrophages | cortical foamy/granular macrophages |  |  |
| cortical vacuolation | cortical vacuolation |  |  |
| decreased vacuolation/cellular eosinophilia of the zona fasciculata | cortical vacuolation |  |  |
| adipocyte accumulation | adipocyte accumulation |  |  |
| decreased zona fasciculata | cortical atrophy |  |  |
| congestion | congestion |  |  |
|  |  |  |  |
|  |  |  |  |
| **brain** |  |  |  |
| focal perivascular inflammation adjacent to the lateral ventricle | inflammation |  |  |
| extramedullary hematopoiesis | extramedullary hematopoiesis |  |  |
| lymphoplasmacytic infiltration of the choroid plexus | infiltrate |  |  |
| vascular congestion | congestion |  |  |
| vacuolation | vacuolation |  |  |
| epithelial vacuolation | epithelial vacuolation |  |  |
|  |  |  |  |
| **muscle,skeletal** |  |  |  |
| degeneration/necrosis | myodegeneration/myonecrosis |  |  |
| hind limb myodegeneration/regeneration | myodegeneration/myoregeneration |  |  |
| myodegeneration | myodegeneration |  |  |
| myopathy | myopathy |  |  |
| myositis | inflammation |  |  |
| hypertrophy of the muscle fiber | hypertrophy |  |  |
|  |  |  |  |
| **heart** |  |  |  |
| increased incidence and severity of the degenerative cardiomyopathy | cardiomyopathy |  |  |
| extramedullary hematopoiesis | extramedullary hematopoiesis |  |  |
| myodegeneration | myodegeneration |  |  |
| myonecrosis | myonecrosis |  |  |
| adipocyte accumulation | adipocyte accumulation |  |  |
| interstitial expansion |  |  |  |
| cardiomyocyte hypertrophy | hypertrophy |  |  |
| distension | distension |  |  |
| myocardial hypertrophy | hypertrophy |  |  |
| brown adipose tissue changes | brown adipose tissue changes |  |  |
| white adipose tissue changes | white adipose tissue changes |  |  |
| cardiomyopathy | cardiomyopathy |  |  |
| mixed cell inflammation in the aorta root | inflammation, infiltrate |  |  |
| vascular congestion | congestion |  |  |
| hypertrophy of the muscle fiber | hypertrophy |  |  |
|  |  |  |  |
| **gland, harderian+gland,lacrimal** |  |  |  |
| degeneration/atrophy | degeneration/atrophy |  |  |
| increased epithelial degeneration/regeneration | epithelial degeneration/regeneration |  |  |
|  |  |  |  |
| **parotid gland+salivary gland+salivary gland,sublingual+ submandibular gland+gland,brunner's** |  |  |  |
| degeneration | degeneration |  |  |
| necrosis | necrosis |  |  |
| atrophy | atrophy |  |  |
| adipocyte accumulation | adipocyte accumulation |  |  |
| enlarged adipocytes | enlarged adipocytes |  |  |
| increase in incidence and severity of acinar vacuolation of the lingual salivary gland | vacuolation |  |  |
| increased secretory content | increased secretory content |  |  |
| mucin depletion in the acinar cells | atrophy |  |  |
| incomplete maturation | incomplete maturation |  |  |
| vacuolated macrophage infiltrate | foamy/granular macrophages |  |  |
|  |  |  |  |
| **Heart** |  |  |  |
| aorta |  |  |  |
| Epicardium |  |  |  |
| macrovacuolation in brown fat | brown adipose tissue vacuolation |  |  |
| adipose tissue atrophy | adipose tissue atrophy |  |  |
|  |  |  |  |
| **mesentery** |  |  |  |
| adipose tissue atrophy | adipose tissue atrophy |  |  |
| white adipose tissue diffuse atrophy | white adipose tissue atrophy |  |  |
|  |  |  |  |
| **stomach** |  |  |  |
| mucosal/submucosal ulcers | erosion/ulceration |  |  |
| inflammation | inflammation |  |  |
| epithelial hyperplasia | epithelial hyperplasia |  |  |
| hyperkeratosis | hyperkeratosis |  |  |
| edema of the submucosa | submucosa edema |  |  |
| enlarged adipocytes | enlarged adipocytes |  |  |
| adipocyte accumulation | adipocyte accumulation |  |  |
| mucosa atrophy | mucosa atrophy |  |  |
| hyperkeratosis | hyperkeratosis |  |  |
| vascular congestion | congestion |  |  |
| glandular erosions | erosion/ulceration |  |  |
| mucosa erosions | mucosa erosions |  |  |
| mucosa hemorrhage | mucosa hemorrhage |  |  |
| lamina propria ulcer | erosion/ulceration |  |  |
| lamina propria necrosis | necrosis |  |  |
|  |  |  |  |
| **prostate gland** |  |  |  |
| incomplete maturation | incomplete maturation |  |  |
| vacuolated macrophage infiltrate | foamy/granular macrophages |  |  |
| apoptosis in the urothelium of the urethra | apoptosis/degeneration urothelium/epithelium?? |  |  |
|  |  |  |  |
| **retroperitoneum+axilla+shoulder** |  |  |  |
| univacuolar fat | white adipose tissue |  |  |
| brown adipose tissue changes | brown adipose tissue changes |  |  |
| increased lymphoid cellularity | hyperplasia |  |  |
| perirenal univacuolar fat | perirenal white adipose tissue |  |  |
|  |  |  |  |
| **bone+sternum** |  |  |  |
| hyperostosis | hyperostosis |  |  |
| intramedullary formation |  |  |  |
| hypocellularity | hypocellularity |  |  |
| hemorrhage | haemorrhage |  |  |
| sinus hyperemia | sinus hyperemia |  |  |
| hyperostosis | hyperostosis |  |  |
| atrophy | atrophy |  |  |
| haemorrhage | haemorrhage |  |  |
|  |  |  |  |
| **bladder+gallbladder** |  |  |  |
| inflammation | inflammation |  |  |
| mixed inflammatory infiltrate | infiltrate |  |  |
| vacuolation | vacuolation |  |  |
| apoptosis | apoptosis/single cell necrosis |  |  |
| urothelium apoptosis, | urothelium apoptosis |  |  |
| urothelium vacuolation, | urothelium vacuolation |  |  |
| vacuolated macrophage infiltrate | foamy/granular macrophages |  |  |
| distension | distension |  |  |
| cholecystitis | inflammation (gall bladder though) |  |  |
| mixed infiltrate | infiltrate |  |  |
| urothelial hyperplasia, | urothelial hyperplasia (or epitheial hyperplasia, bladder) |  |  |
| uroliths | uroliths |  |  |
|  |  |  |  |
| **vagina** |  |  |  |
| increased mucification | increased mucification |  |  |
|  |  |  |  |
| **oral cavity+tongue** |  |  |  |
| ulceration in the mucosa | mucosa erosion/ulceration |  |  |
| single cell necrosis (mucosal epithelium) | mucosa epithelial single cell necrosis |  |  |
| mixed infiltrates | infiltrate |  |  |
|  |  |  |  |
| **Adipose Tissue, Brown** |  |  |  |
| macrovacuolar (unilocular) changes, | vacuolation |  |  |
| increased and enlarged adipocytes | adipocyte accumulation, enlarged adipocytes |  |  |
|  |  |  |  |
| **ureter** |  |  |  |
| dilatation | dilatation |  |  |
| mixed cell infiltrates | infiltrate |  |  |
|  |  |  |  |
| **nasal turbinate** |  |  |  |
| brown pigment in the olfactory epithelium | pigment (epithelium) |  |  |
|  |  |  |  |
| **mucosa** |  |  |  |
| lamina propria ulcer | lamina propria ulcer |  |  |
| lamina propria necrosis | lamina propria necrosis |  |  |

**Table.S3.txt** and **S4.txt** are provided as separate files in the GitHub repository <https://github.com/Roche/CSL-Tox> due to the size of the datasets.

**Table.S5A** Details on dose level adjustments between short-term and long-term studies.

| **Compound** | **Doses tested*** | **Dose adjustments** |
| --- | --- | --- |
| Compound 8 | 0,5,20,60 in 39-w,13-w (marmoset) &  0,2,8 in 4-w (dog) | No |
| Compound 9 | 0,30,100, 300 mg/kg/day (4-w, 16-w & 26-w rat)  **0,25,50,100** for 4-w dog  **0,12.5, 25** for 13-w & 39-w dog | Yes  (in non-rodents) |
| Compound 10 | 0,30,100,300 (4-w,13-w & 29-w dog ) &  0,50,150,500 (4-w,13-w & 26-w rat) | No |
| Compound 11 | **0,0.05,0.3,1** (8-w cyno)  **0,0.15,0.05,0.1** (39-w cyno)  **0.1,0.3,1,3** (13-w rat)  **0.1,0.25,0.5** (26-w & 39-w rat) | Yes (in rodents and non-rodents) |

*Dose units = mg/kg/day

Details on the dose adjustments:

Compound-8

For compound 8 the dose levels have not been adjusted. Compound 8 was tested in two non-rodent species. The short-term study (4-week) was performed in dog and dogs did not tolerate the compound (responded with vomiting and body weight loss). Therefore the non-rodent species was changed to marmoset and higher doses could be tested in marmoset where no dose adjustments were performed.

Compound-9

For compound 9 dose levels were adjusted: Compound 9 caused convulsions at the highest dose (100 mg/kg) tested in the 4-w dog study. No convulsions, but transient clinical signs and effects on RBC and reticulocytes were observed at the next lower dose of 50 mg/kg in that study. Therefore the NOAEL was set at 25 mg/kg.

In the 13-w and 9-m study 50 mg/kg was the highest dose tested and led to clinical signs, (vomiting, salivation and decreased activity). Hematological effects were no longer observed in these studies and the NOAEL was set at 50 mg/kg. The increase in the NOAEL with increased duration of treatment may have been caused by some kind of tolerance induction to the effect of the compound on erythropoiesis.

Compound-10

No dose adjustments for rodents or non-rodents, where same doses were tested in all durations for both species (dog and rat).

Compound-11

Doses levels were decreased due to transient clinical signs and clinical pathology changes in rodents and non-rodents.

**Table.S5B** Discrepancies in the NOAEL changes outcome between the algorithm and the toxicologist interpretation.

|  | **Species** | **Compounds** | **Algorithm** | **Toxicologist interpretation** |
| --- | --- | --- | --- | --- |
| **Small molecules** | **Rodents** | Compound-9  Compound-25 | Decrease | Same |
|  | **Non-Rodents** | Compound-8 | Increase | Same |
|  |  | Compound-21 | Increase | Decrease |
| **Large molecules** | **Rodents** | Compound-K | Increase | Same |
|  | **Non-rodents** | Compound-K | Increase | Same |
|  |  | Compound-Q | Decrease | Same |
